# Clusterin facilitates glioma progression via BCL2L1-dependent regulation of apoptotic resistance

**DOI:** 10.3389/fnmol.2025.1596021

**Published:** 2025-06-18

**Authors:** Qingqing Xu, Xin Liu, Yibo Zhang, Shiyu Yuan, Wenli Huang, Mingshan Pi, Qi Xiong, Hongyan Zhou, Yuran Gui, Yifan Xiao, Xiaochuan Wang, Xiji Shu, Yiyuan Xia

**Affiliations:** ^1^Hubei Key Laboratory of Cognitive and Affective Disorders, Jianghan University, Wuhan, China; ^2^School of Medicine, Institutes of Biomedical Sciences, Jianghan University, Wuhan, China; ^3^Department of Pathology and Pathophysiology, School of Medicine, Jianghan University, Wuhan, China; ^4^Hubei Provincial Demonstration Center for Experimental Medicine Education, School of Medicine, Jianghan University, Wuhan, China

**Keywords:** glioma, clusterin (CLU), migration, proliferation, BCL2L1, apoptosis

## Abstract

**Background:**

Clusterin (CLU) is a multifunctional protein involved in various pathophysiological processes and diseases. Glioma, the most common aggressive primary brain tumor, is characterized by high morbidity, mortality, and extremely poor prognosis. Our research has found that CLU is upregulated in glioma and contributes to increased tumor malignancy. However, the specific regulatory mechanisms of CLU in the context of glioma are not fully understood.

**Methods:**

We used glioma public databases, immunohistochemistry (IHC), and immunoblotting techniques to evaluate the expression levels and prognostic value of CLU in glioma. Cell migration and proliferation assays, including the scratch wound healing and MTT assays, were conducted to assess the functional impact of CLU. In addition, immunoblotting and flow cytometry were used to analyze apoptosis-related proteins and CLU-BCL2L1 interactions. An *in situ* tumor model using nude mice was established to investigate the effects of CLU *in vivo*.

**Results:**

Bioinformatics analyses showed that CLU was highly expressed in glioma, associated with poor clinical outcomes. Functional assays revealed that CLU and BCL2L1 promoted glioma cell migration and proliferation. Silencing CLU reduced the migration and proliferation of glioma cells, while overexpression of CLU enhanced these aggressive phenotypes. Mechanistic studies showed CLU regulated BCL2L1 expression, inhibiting apoptosis pathways and promoting malignancy. *In vivo* experiments confirmed the inhibitory effects of CLU downregulation on glioma growth.

**Conclusion:**

This study clarifies the role of the CLU-BCL2L1 axis in promoting glioma migration and proliferation both *in vitro* and *in vivo*. It suggests that targeting this pathway may be a promising therapeutic strategy for glioma.

## Introduction

Gliomas, the most common primary malignant brain tumors, account for 40–60% of all intracranial neoplasms and are classified by the World Health Organization (WHO) into grades I–IV based on histopathological features and molecular markers (Weller et al., 2024). Despite advancements in surgical techniques, radiotherapy, and chemotherapy, the prognosis remains poor, particularly for high-grade gliomas such as glioblastoma (GBM), with a 5-year survival of 7.2% (Wu et al., 2021). The diagnosis of gliomas primarily relies on imaging techniques such as magnetic resonance imaging (MRI), which has limitations in detecting early stage tumors and differentiating tumor subtypes (Youssef and Wen, 2024). Histopathological examination and genetic analysis are also crucial for accurate diagnosis, but they are invasive and may not always be feasible (Jiang et al., 2017). Recent progress has shifted toward precision medicine, including targeted therapies and immunotherapies. For instance, “Cancer neuroscience,” an emerging convergent field in biomedicine, investigates the dynamic bidirectional interplay between tumorigenesis and neural signaling networks, bridging oncology, neurobiology, and systems biology (Huang et al., 2025). Additionally, the discovery of extracellular vehicles (EVs) has opened up new possibilities for non-invasive diagnosis. EVs released by glioma cells contain various proteins and nucleic acids that can be used as biomarkers and therapeutic tools (Cela et al., 2024). Novel strategies such as CAR-T cell therapy and tumor-treating fields (TTFields) are also under investigation to improve therapeutic outcomes (Giotta Lucifero and Luzzi, 2022).

The aggressiveness of gliomas is driven by a complex interplay of genetic, epigenetic, and microenvironmental factors: Mutations in IDH1/2, TP53, and EGFR are strongly associated with tumorigenesis and progression. For example, IDH1 mutations activate hypoxia-inducible factor 1α (HIF-1α), promoting angiogenesis and metabolic reprogramming (Huang et al., 2019). Tumor-associated macrophages (TAMs) and extracellular matrix (ECM) remodeling facilitate invasion by secreting matrix metalloproteinases (MMPs) and cytokines that degrade the blood-brain barrier (BBB) (Yu et al., 2024). Hypoxic niches further drive adaptive responses, including epithelial-mesenchymal transition (EMT), which enhances migratory capacity (Hapke and Haake, 2020).

Apoptosis, or programmed cell death, is a critical mechanism for maintaining tissue homeostasis and eliminating damaged or infected cells. In gliomas, the dysregulation of apoptosis pathways contributes to treatment resistance. The B-cell lymphoma 2 (BCL-2) family of proteins plays a central role in regulating apoptosis. Overexpression of anti-apoptotic proteins like BCL-2 and BCL-xL in glioma cells can inhibit apoptosis and promote treatment resistance (Golla et al., 2021). The p53 tumor suppressor gene is another key player in apoptosis. Mutations in p53 are frequently found in gliomas and can lead to the loss of its tumor suppressor function, resulting in increased cell proliferation and resistance to apoptosis (Muller et al., 2009). Additionally, the activation of survival pathways like the PI3K/Akt/mTOR pathway can further inhibit apoptosis and enhance treatment resistance in gliomas (Muller et al., 2009).

Clusterin (CLU) is a glycoprotein involved in various cellular processes, including cell proliferation, DNA damage repair, and apoptosis. It has been shown to be overexpressed in many cancers, including gliomas, and is associated with poor prognosis (Praharaj et al., 2021). The role of CLU in apoptosis is complex and context-dependent. In some cases, CLU can promote apoptosis by interacting with pro-apoptotic proteins like BAX and Bak, leading to the release of cytochrome c and the activation of caspases (Liu et al., 2021). In other cases, CLU can inhibit apoptosis by interacting with anti-apoptotic proteins like BCL-2 and BCL-xL (Wang et al., 2012). Recent studies have revealed that CLU is involved in the progression of gliomas. Downregulation of CLU has been shown to inhibit cell proliferation and induce cellular senescence in astrocyte cells, suggesting that CLU may play a role in maintaining the viability and growth of glioma cells (Sultana et al., 2024). Additionally, CLU has been found to be associated with the expression of pro-inflammatory cytokines like IL-1β, which can contribute to the development of a senescent phenotype in glioma cells (Ungsudechachai et al., 2022; Fathima Hurmath et al., 2014).

Given the role of CLU in apoptosis and its association with cancer progression, it is plausible that CLU may influence the malignancy of gliomas through apoptosis pathways. Downregulation of CLU has been shown to induce cellular senescence and inhibit cell proliferation in cancer cells, suggesting that targeting CLU could be a potential therapeutic strategy for gliomas (Mitsufuji et al., 2022). Additionally, the involvement of CLU in the regulation of pro-inflammatory cytokines and the senescent phenotype indicates that it may play a role in the tumor microenvironment and contribute to the aggressiveness of gliomas (Yang et al., 2023; Zhang et al., 2023). In this study, we clarify the role of the CLU-BCL2L1 axis in promoting glioma migration and proliferation via apoptotic resistance both *in vitro* and *in vivo*. It suggests that targeting this pathway may be a promising therapeutic strategy for glioma.

## Results

### Positive association between CLU expression and histological grades of glioma in patients

**FIGURE 1:**
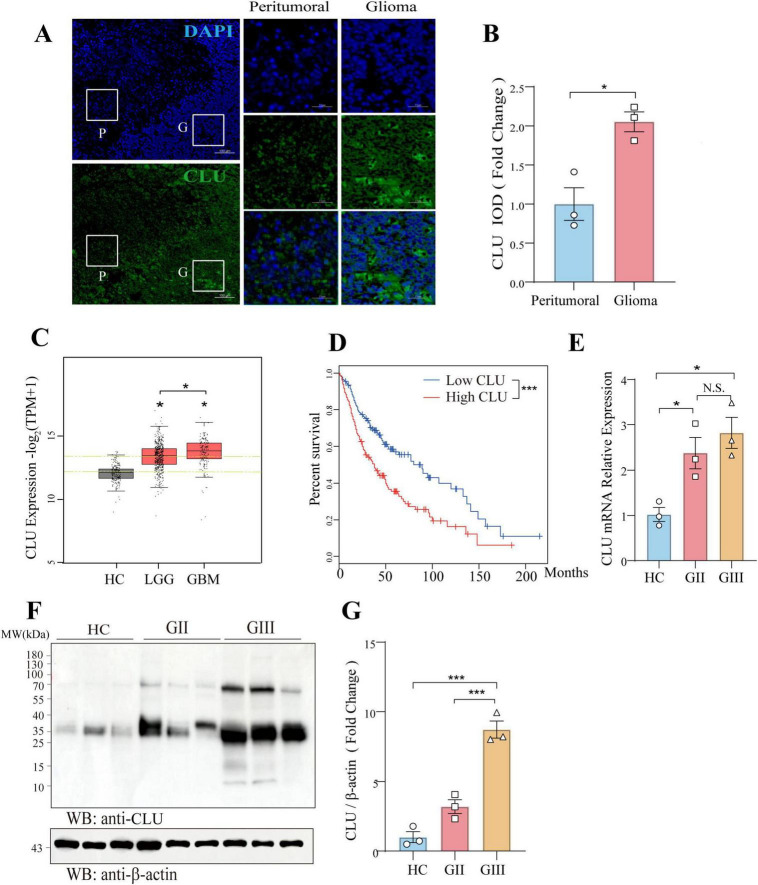
CLU is positively associated with glioma in patients. **(A)** Representative image of CLU (green) and DAPI (blue) in pathological brain tissue from glioma patients (scale bar, 100 μm). **(B)** Expression of CLU in pathological brain tissue from glioma patients was analyzed (*n* = 3 biologically independent samples per group). Data are normalized to the peritumoral region and presented as mean ± SEM. Statistical analysis was performed using an unpaired two-tailed Student’s *t*-test, *p < 0.05. **(C)** CLU mRNA expression levels in glioma patients were analyzed. Data are normalized to the healthy control (HC) group and presented as mean ± SEM. Statistical analysis was performed using an unpaired two-tailed Student’s *t*-test, *p < 0.05. **(D)** Survival percentages of glioma patients with high and low CLU levels were analyzed. Data are presented as mean ± SEM. Statistical analysis was performed using a two-tailed Mann-Whitney U test (non-parametric test), ***p < 0.001. **(E)** CLU mRNA levels in glioma tissues of different grades were analyzed using real-time fluorescence quantitative PCR (*n* = 3 biologically independent samples per group). Data are normalized to the HC group and presented as mean ± SEM. Statistical analysis was performed using a one-way ANOVA test, *p < 0.05. **(F,G)** CLU protein levels in glioma tissues of different grades were analyzed using Western blot (*n* = 3 biologically independent samples per group). Data are normalized to the HC group and presented as mean ± SEM. Statistical analysis was performed using a one-way ANOVA test, ***p < 0.001.

To elucidate the potential role of CLU in glioma, we examined its expression in glioma tissues using immunofluorescence on pathological brain samples from patients with glioma. The fluorescence intensity of CLU protein was significantly higher in the glioma region compared to the peritumoral region (Figures 1A,B), suggesting a possible promoting role of CLU in glioma progression. Based on the Gepia2.0 database (integrating TCGA-LGG/GBM and GTEX normal brain tissue data), the data we used were from TCGA-LGG/GBM (dbGaP Study Accession phs000178) and Genotype-Tissue Expression Project (GTEx-Brain). The CLU mRNA expression in 519 LGG, 163 GBM and 208 normal brain tissues was analyzed, the level of CLU in tumor tissues was significantly higher than that in normal tissue. Compared to healthy controls (HC), CLU mRNA expression was significantly elevated in patients with lower-grade glioma (LGG, WHO grades II and III) and glioblastoma (GBM, WHO grade IV) (Figure 1C). Notably, CLU mRNA levels were higher in GBM than in LGG. Consistent with these findings, we observed a negative correlation between CLU levels and survival rates, with patients exhibiting high CLU expression having a significantly lower survival rate (Figure 1D). Additionally, we validated these results using pathological brain tissue from glioma patients. The mRNA (Figure 1E) and protein (Figures 1F,G) levels of CLU were consistently higher in the glioma region compared to the HC group. Specifically, CLU levels in grade III glioma (GIII) were significantly higher than those in grade II glioma (GII). Collectively, these findings demonstrate a positive association between CLU expression and glioma in patients.

### Modulation of CLU expression regulates migration and proliferation in glioma cell lines

**FIGURE 2:**
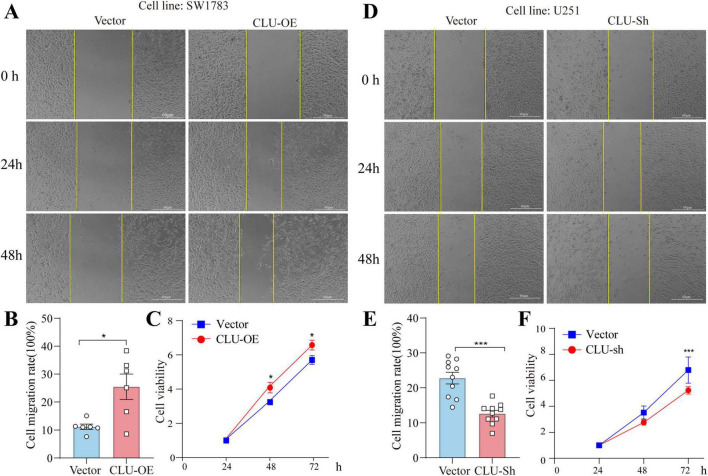
CLU intervention regulates the migration and growth of glioma cell lines. **(A)** Representative images of scratch assays from SW1783 cell lines with or without CLU overexpression. **(B)** Cell migration rates were analyzed after CLU overexpression (*n* = 6 biologically independent samples per group). Data are normalized to the vector control group and presented as mean ± SEM. Statistical analysis was performed using an unpaired two-tailed Student’s *t*-test, *p < 0.05. **(C)** Cell growth rates were analyzed after CLU overexpression using the MTT assay (*n* = 5 biologically independent samples per group). Data are normalized to the 24-h time point and presented as mean ± SEM. Statistical analysis was performed using a two-way ANOVA test, *p < 0.05. **(D)** Representative images of scratch assays from U251 cell lines with or without reduced CLU expression. **(E)** Cell migration rates were analyzed after CLU knockdown (*n* = 10 biologically independent samples per group). Data are normalized to the vector control group and presented as mean ± SEM. Statistical analysis was performed using an unpaired two-tailed Student’s *t*-test, *p < 0.05. **(F)** Cell growth rates were analyzed after CLU knockdown using the MTT assay (*n* = 5 biologically independent samples per group). Data are normalized to the 24-h time point and presented as mean ± SEM. Statistical analysis was performed using a two-way ANOVA test, ***p < 0.001.

To further investigate the role of CLU in glioma, we conducted a series of experiments using various glioma cell lines. We observed that the protein levels of CLU varied among different cell lines, with the highest expression in the U251 cell line and the lowest in the SW1783 cell line (Supplementary Figures S1A,B). Based on these findings, we selected specific glioma cell lines for our subsequent experiments. First, we overexpressed CLU in the SW1783 cell line. As shown in the results (Supplementary Figures 2A,B), both CLU mRNA and protein levels increased significantly after transfection with the CLU plasmid, confirming the successful overexpression. Compared to the vector control group, cells overexpressing CLU exhibited enhanced migration and proliferation (Figures 2A–C). Conversely, we efficiently knocked down CLU expression in the U251 cell line (Supplementary Figures S2C,D). Consistent with the overexpression results, cells with reduced CLU expression (CLU-sh group) exhibited significantly slower migration and proliferation compared to the vector control group (Figures 2D–F). We also successfully knocked down CLU expression in the U87 cell line (Supplementary Figures S3A,B), and similarly observed that cells in the CLU-sh group migrated and proliferated more slowly than the vector control group (Supplementary Figures S3C,E). Overall, these results demonstrate that CLU promotes glioma progression primarily by accelerating cell migration and proliferation.

### CLU upregulates the apoptosis-related gene BCL2L1 in glioma cell lines

To elucidate the protein-protein interactions involving CLU and its related genes, we employed the STRING online tool to construct a protein-protein interaction (PPI) network (Supplementary Figure S4A). In this network, CLU occupies a central position, interacting with multiple genes, including BCL2L1, BAX, and XRCC6. The close connectivity of these genes to CLU suggests potentially strong interactions. Additionally, several gene modules are evident within the network. For instance, genes associated with heat-shock proteins (HSPA5, HSP90AA1, HSP90B1) are interconnected, indicating that they may function synergistically in specific biological processes. These findings provide valuable insights into the functional mechanisms of CLU in relevant physiological and pathological contexts, and further experimental validation of these interactions is warranted.

To further investigate the mechanisms underlying CLU’s role in glioma, we utilized two bioinformatics tools, TIMER2.0 (Supplementary Figure S4B–I) and GEPIA 2.0 (Supplementary Figure S4J–Q), to analyze the correlations between CLU expression and the expression of BAX, BCL2L1, PRNP, HSPA5, XRCC6, APP, LRP2, and TTR genes. According to the TIMER2.0 database analysis (Supplementary Figure S4B–I), several genes (BAX, BCL2L1, PRNP, HSPA5, LRP2, TTR, all *p* < 0.05) exhibited positive correlations with CLU expression. XRCC6 showed a negative correlation, while APP displayed no significant correlation with CLU. These results suggest that these genes may interact synergistically or antagonistically with CLU during glioma development. Similar correlation trends were also observed in the GEPIA 2.0 analysis (Supplementary Figure S4J–Q), further supporting their underlying role in glioma.

**FIGURE 3:**
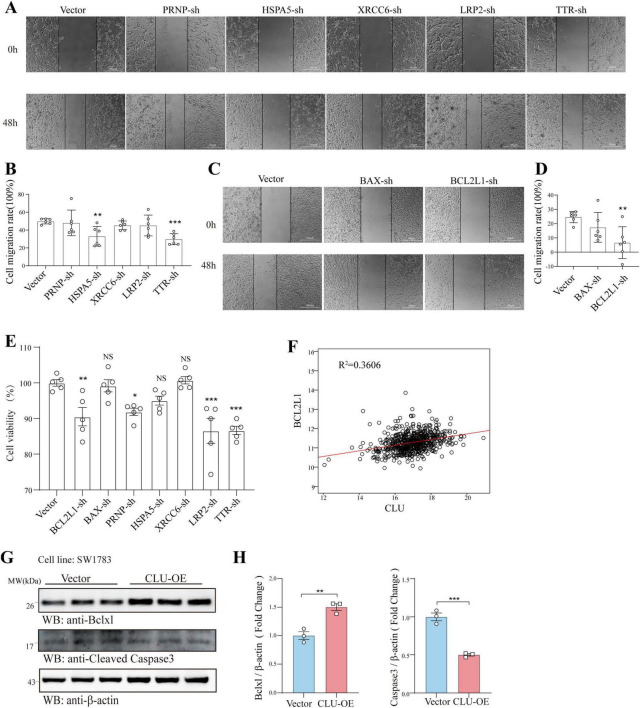
CLU regulates the apoptosis-related gene BCL2L1 in glioma cell line. In U251 cells, the genes PRNP, HSPA5, XRCC6, LRP2, TTR, BAX, and BCL2L1 were individually knocked down. **(A–D)** The migration of glioma cells was assessed using scratch assays following the knockdown of each gene (*n* = 6 biologically independent samples per group). **(E)** The effects of knocking down these genes on glioma cell proliferation were analyzed using MTT assays (*n* = 5 biologically independent samples per group). **(F)** The correlation between CLU and BCL2L1 was analyzed using data from glioma patients. **(G)** The expression of BCL2L1 and cleaved caspase-3 was detected by Western blot in the SW1783 cell line after CLU overexpression (*n* = 3 biologically independent samples per group). **(H)** Data are normalized to the vector control group and presented as mean ± SEM. Statistical analysis was performed using an unpaired two-tailed Student’s *t*-test, *p < 0.01, ***p < 0.001.

To furtherly explore the role of the seven candidate genes (PRNP, HSPA5, BCL2L1, etc.) in glioma, we next verified their role using scratch and MTT experiments in U251 cells. In scratch assay, HSPA5, TTR, and BCL2L1 knocking down significantly inhibited cell migration (Figures 3A–D). While in MTT experiment, BCL2L1, PRNP, LRP2, and TTR knocking down significantly inhibited cell proliferation (Figure 3E). Together, both BCL2L1 and TTR knocking down significantly inhibited cell migration and proliferation rate. Given that BCL2L1 (promoting cell survival) is closely linked to promoting tumor progression, we explored its underlying mechanisms related to CLU. Analysis of glioma patient data revealed a positive correlation between CLU and BCL2L1 expression (Figure 3F). To verify this relationship, we overexpressed CLU in the SW1783 cell line and measured BCL2L1 expression. The results showed that CLU overexpression significantly upregulated BCL2L1 while inhibiting cleaved caspase-3, a downstream apoptotic pathway signal, compared to the vector control group (Figures 3G,H).

However, the specifically regulation role of CLU for BCL2L1 is still unknown. Then we explored it at the level of mRNA transcription and proteins physically interaction. The result displayed that in SW1783, the mRNA level of BCL2L1 in CLU-OE group was increased suggesting the regulation role of CLU for BCL2L1 at the transcriptional level (Supplementary Figure S5A). On the one hand, CLU protein and BCL2L1 protein were not interacted physically as showed in the result of co-immunoprecipitation (Supplementary Figure S5B).

### Inhibition of BCL2L1 reverses the pro-tumorigenic effects of CLU in glioma cell lines

**FIGURE 4:**
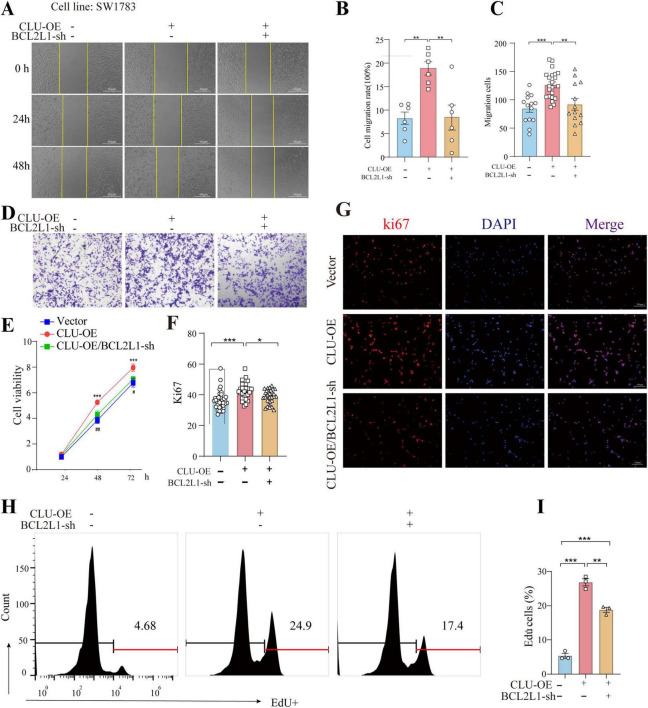
BCL2L1 inhibition reverses the pro-tumor proliferation and migration effects of CLU in glioma cell lines. **(A)** Representative images of scratch assays from SW1783 cell lines with or without CLU overexpression (CLU-OE) or BCL2L1 knockdown (BCL2L1-sh). **(B)** Cell migration rates were analyzed following CLU-OE or BCL2L1-sh treatment (*n* = 6 biologically independent samples per group). Data are normalized to the empty vector group and presented as mean ± SEM. Statistical analysis was performed using a one-way ANOVA test, **p < 0.01. **(C)** Graphic representation of migrated cells counts from Transwell assay. Data are represented as the mean ± SEM from three independent experiments. **p < 0.01; ***p < 0.001, relative to control. **(D)** Representative images of Transwell migration assays performed. **(E)** Cell growth rates were analyzed following CLU-OE or BCL2L1-sh treatment using the MTT assay (*n* = 5 biologically independent samples per group). **(F)** Representative image of Ki67 (red) and DAPI (blue) in the SW1783 cell line (scale bar, 100 μm). **(G)** The percentage of EdU+ cells. Data are normalized to the empty vector group and presented as mean ± SEM. Statistical analysis was performed using a one-way ANOVA test, *p < 0.05 and ***p < 0.001. **(H)** Representative images of EdU detected by flow cytometry. **(I)** The percentage of EdU+ cells was measured by flow cytometry. Data are normalized to the empty vector group and presented as mean ± SEM. Statistical analysis was performed using a one-way ANOVA test, **p < 0.01 and ***p < 0.001.

**FIGURE 5:**
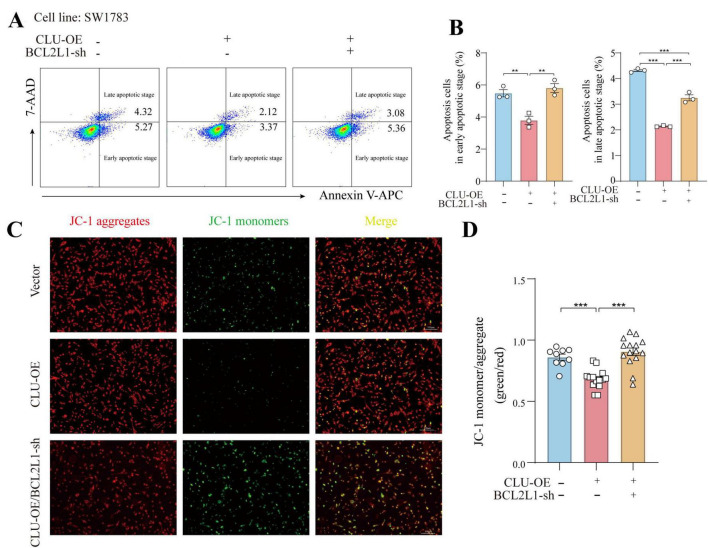
CLU promotes glioma development through BCL2L1 anti-apoptotic effect. **(A)** Representative images of early and late apoptotic cells detected by flow cytometry. **(B)** The percentage of early and late apoptotic cells was measured by flow cytometry. Data are normalized to the empty vector group and presented as mean ± SEM. Statistical analysis was performed using a one-way ANOVA test, **p < 0.01 and ***p < 0.001. **(C)** Fluorescence micrographs of ΔψM of SW1783 cells (scale bar = 100 μm). ΔψM was observed after JC-1 staining. Micrographs represented 3 independently replicate experiments. **(D)** Quantifications of ΔψM. Data are normalized to the empty vector group and presented as mean ± SEM. Statistical analysis was performed using a one-way ANOVA test, ***p < 0.001.

The findings above established a connection between CLU and BCL2L1, but whether CLU promotes glioma via BCL2L1 remained unclear. To address this, we simultaneously overexpressed CLU and knocked down BCL2L1 in the SW1783 cell line. The results indicated that both CLU overexpression and BCL2L1 knockdown were successfully achieved (Supplementary Figures S6A,B). In terms of cell migration, CLU overexpression significantly enhanced cell migration compared to the vector control group. However, this effect was reversed by BCL2L1 knockdown. The results were consistent in wound-healing experiment (Figures 4A,B) and transwell assay (Figures 4C,D). Similarly, the growth-promoting effect of CLU overexpression was attenuated by BCL2L1 knockdown, as evidenced by slower cell proliferation in the CLU-overexpressing group when BCL2L1 was reduced using MTT assay (Figure 4E). Moreover, Ki67 (Figures 4F,G) and EdU staining (Figures 4H,I) for cell proliferative also displayed the same conclusion. We also examined the impact on cell apoptosis and found that both early and late apoptotic cells were decreased in CLU-overexpressing cells compared to the vector control group. This effect was reversed by BCL2L1 knockdown (Figures 5A,B). Moreover, the mitochondrial membrane potential detection (JC-1 staining) also showed that the loss of mitochondrial transmembrane potential (ΔΨm) in the CLU-OE group was significantly decreased indicating reduced cell apoptosis, which was restored after BCL2L1 knockdown (Figures 5C,D).

Collectively, these results suggest that CLU accelerates cell migration and growth while inhibiting apoptosis in glioma cell lines primarily through the upregulation of BCL2L1.

### Down regulation of BCL2L1 inhibits CLU-induced tumor formation in nude mice

To further validate the conclusions drawn from our *in vitro* experiments, we used orthotopic transplantation models and subcutaneous tumor xenograft models. We inoculated the brain and skin of these mice with SW1783 cells that had been stably transfected with either the CLU-overexpression (CLU-OE) virus or the BCL2L1 short-hairpin RNA (BCL2L1-sh) virus. The successful overexpression of CLU and knockdown of BCL2L1 were confirmed (Supplementary Figures S7A,B).

**FIGURE 6:**
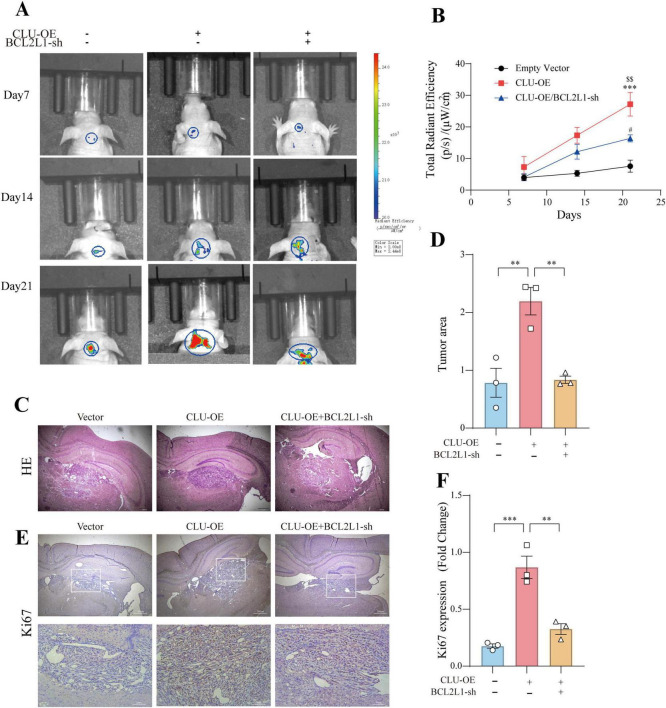
BCL2L1 down regulation inhibits glioma formation promoted by CLU in the brain of nude mice. **(A)** Representative tumor bioluminescence images of mice at 7, 14, and 21 days after tumor implantation in an orthotopic xenograft model generated by SW783 cells transfected with an empty vector, CLU overexpression, or BCL2L1 knock-down. **(B)** Tumor volume was measured every 7 days for 21 days following subcutaneous inoculation of the SW1783 cell line in nude mice (*n* = 3 biologically independent samples per group). Data are presented as mean ± SEM and analyzed using a two-way ANOVA test. Statistical significance is indicated as follows: ***p < 0.001 for empty vector vs. CLU-OE; ^$$^*p* < 0.001 for CLU-OE vs. BCL2L1-sh; ^#^*p* < 0.05 for empty vector vs. BCL2L1-sh. **(C)** H&E staining of sections from mouse brains with SW1783 xenografts at 21 days after implantation. **(D)** Calculation of the growth area of orthotopic transplanted tumors by HE staining. Data (*n* = 3 biologically independent samples per group) are presented as mean ± SEM and analyzed using a one-way ANOVA test, **p < 0.01. **(E)** Representative images of immunohistochemical staining for Ki67 in the tumors of nude mice. **(F)** The number of proliferating cells (Ki67-positive) was analyzed by immunohistochemistry. Data (*n* = 3 biologically independent samples per group) are presented as mean ± SEM and analyzed using a one-way ANOVA test, **p < 0.01, ***p < 0.001.

Tumors derived from CLU-overexpressing SW1783 cells grew more rapidly compared to those from the vector control group (Figures 6A,B; Supplementary Figures S8A,B). Conversely, when BCL2L1 was knocked down in the SW1783 cell line, tumor growth was significantly inhibited compared to the CLU-OE group. Consistent with these observations, tumor weight was higher in the CLU-overexpression group than in the vector control group, while tumors from the BCL2L1-sh group were lighter than those from the CLU-OE group (Supplementary Figure S8C). Histological analysis via hematoxylin and eosin (H&E) staining revealed that tumor tissues from the CLU-OE group exhibited more pronounced cellular heteromorphism compared to those from the vector control group (Figures 6C,D; Supplementary Figure 8D). These heteromorphic features included larger nuclei, more intensely stained chromatin, and a more disordered tissue architecture. However, these features were attenuated in tumors from the BCL2L1-sh group compared to the CLU-OE group. Immunohistochemical analysis further indicated that the number of proliferating cells was higher in the CLU-OE group than in the vector control group. In contrast, BCL2L1 knockdown significantly reduced the number of proliferating cells compared to the CLU-OE group (Figures 6E,F; Supplementary Figure S8E). Collectively, these *in vivo* results demonstrate that CLU promotes glioma formation, with its effects mediated through the upregulation of BCL2L1.

## Discussion

Gliomas, the most common primary brain tumors, are characterized by their aggressive nature and poor prognosis. Despite advancements in surgical resection, radiotherapy, and chemotherapy, the recurrence and progression of gliomas remain inevitable, highlighting the need for novel therapeutic targets and biomarkers. This discussion focuses on the role of clusterin in gliomas, particularly its expression patterns, and prognostic significance, as well as its potential as a therapeutic target.

Clusterin, a multifunctional chaperone protein, plays context-dependent roles in tumorigenesis. Physiologically, it regulates protein homeostasis, apoptosis, and cell cycle progression (Ren et al., 2023). CLU expression has been found to be significantly upregulated in gliomas compared to normal brain tissue. This upregulation is more pronounced in higher-grade gliomas, such as glioblastoma multiforme (GBM), compared to lower-grade gliomas (LGG) (Ren et al., 2023). The elevated expression of CLU in gliomas suggests its potential role in tumor progression and malignancy. Studies have shown that CLU expression is associated with various clinicopathological features, including tumor grade and patient survival outcomes (Ostrom et al., 2022).

High CLU expression in LGG patients is associated with worse overall survival (OS), disease-specific survival (DSS), and progression-free interval (PFI) (Schaff and Mellinghoff, 2023). Univariate and multivariate Cox regression analyses have identified CLU expression as an independent risk factor for the prognosis of LGG patients, with a hazard ratio of 1.451 (Rodriguez-Camacho et al., 2022). These findings indicate that CLU expression can serve as a prognostic biomarker for LGG, providing valuable information for clinical management and treatment decisions.

In human osteosarcoma cells, CLU overexpression correlates with therapeutic resistance. Secretory CLU (sCLU) binds to Bax and inhibits mitochondrial apoptosis (Trougakos et al., 2009), while intracellular CLU (iCLU) promotes oxidative stress adaptation (Trougakos et al., 2005). Conversely, CLU knockdown sensitizes osteosarcoma cells to cisplatin, suggesting its dual role as a stress-responsive cytoprotectant and metastasis promoter (Huang et al., 2014). While CLU is generally associated with tumor progression and poor prognosis in gliomas and other cancers, its role can vary depending on the tumor type and genetic context. For instance, CLU has been identified as a tumor suppressor in neuroblastomas (Chayka et al., 2009). These findings position CLU as a potential therapeutic target, though its pleiotropic effects necessitate context-specific modulation strategies.

This study comprehensively investigated the role of CLU in glioma progression by examining its expression in glioma tissues and cell lines, as well as its impact on tumor formation *in vivo*. Firstly, immunofluorescence analysis revealed that CLU protein levels were significantly higher in glioma regions compared to peritumoral regions (Figures 1A,B). Further analysis of CLU mRNA expression in a database showed elevated levels in patients with lower-grade glioma (LGG) and glioblastoma (GBM) compared to healthy controls (HC) (Figure 1C). Notably, CLU mRNA levels were higher in GBM than in LGG. A positive correlation was observed between CLU levels and survival rates, with high CLU expression associated with significantly lower survival rates (Figure 1D). These findings were validated using pathological brain tissue from glioma patients, demonstrating higher mRNA and protein levels of CLU in glioma regions compared to HC, with grade III glioma (GIII) showing significantly higher CLU levels than grade II glioma (GII) (Figures 1E,F).

*In vitro* experiments using glioma cell lines (U251, SW1783, U87) demonstrated that CLU overexpression enhances cell migration and proliferation, while CLU knockdown inhibits these processes (Figures 2A–F; Supplementary Figures S2A,B, 3A–E). These results highlight CLU’s role in promoting glioma progression by accelerating cell migration and proliferation. Bioinformatics analysis using TIMER2.0 (Supplementary Figures S4B–I) and GEPIA 2.0 (Supplementary Figures S4J–Q) revealed positive correlations between CLU expression and several genes (BAX, BCL2L1, PRNP, HSPA5, LRP2, TTR, all *p* < 0.05), suggesting synergistic or antagonistic interactions during glioma development. As for the seven candidate genes (PRNP, HSPA5, BCL2L1, etc.), both scratch and MTT experiments showed that only BCL2L1 knocking down significantly inhibited cell migration and proliferation rate in U251 cells, while other genes knockdown groups showed only weak inhibition or no significant difference (Figures 3A–E). The result suggested that BCL2L1 was a key downstream molecule of the pro-cancer effect of CLU. Further experiments showed that CLU overexpression in SW1783 cells significantly upregulated BCL2L1 and inhibited cleaved caspase-3, a downstream apoptotic signal (Figures 3G–H). Inhibiting BCL2L1 reversed the pro-migratory and pro-proliferative effects of CLU overexpression and restored apoptosis in glioma cells (Figures 4A–E, 5A–D). These findings suggest that CLU accelerates cell migration and growth while inhibiting apoptosis primarily through the upregulation of BCL2L1.

*In vivo* studies in nude mice further validated these findings. CLU overexpression promoted tumor growth, which was significantly inhibited by BCL2L1 knockdown (Figures 6A,B; Supplementary Figures 8A,B). Histological and immunohistochemical analyses confirmed the role of CLU in tumor formation through BCL2L1 upregulation (Figures 6C,D; Supplementary Figures S8C,D). Collectively, these results demonstrate that CLU promotes glioma formation *in vivo*, with its effects mediated through the upregulation of BCL2L1.

The current study’s findings are based on experiments conducted in nude mice, which provided valuable insights into the role of CLU and BCL2L1 in glioma progression. However, the use of more diverse animal models, such as genetically engineered mice or patient-derived xenografts, could offer a more comprehensive understanding of CLU’s role in glioma progression. In addition, the study identified CLU and BCL2L1 as key players in glioma progression, but the detailed molecular mechanisms underlying their interactions remain to be fully elucidated. The complex interplay between CLU, BCL2L1, and other signaling pathways may vary across different glioma subtypes and genetic backgrounds. Future studies should explore additional targets within the CLU-BCL2L1 axis, such as upstream regulators and downstream effectors. For example, investigating the role of CLU in modulating other anti-apoptotic proteins or signaling pathways could provide new therapeutic insights. Moreover, the development of targeted therapies, such as antisense oligonucleotides (ASOs) or small interfering RNAs (siRNAs), could offer selective and effective means of downregulating CLU and BCL2L1. Preclinical studies have shown promise in using ASOs to reduce CLU levels, suggesting a potential avenue for glioma treatment (Mamun et al., 2024).

In summary, our study provides compelling evidence that CLU plays a significant role in glioma progression by enhancing cell migration and proliferation, while inhibiting apoptosis primarily through the upregulation of BCL2L1. Elevated CLU expression in glioma tissues correlates with advanced tumor grades and poorer patient survival, highlighting its potential as a prognostic biomarker. Furthermore, both *in vitro* and *in vivo* experiments confirm that CLU’s pro-tumorigenic effects are mediated by BCL2L1, suggesting that targeting this pathway could be a promising therapeutic strategy for glioma treatment. Future research should focus on elucidating the detailed molecular mechanisms underlying CLU-BCL2L1 interactions and exploring the potential of CLU-targeted therapies in clinical settings.

## Materials and methods

### Bioinformatics analysis

CLU mRNA expression in glioma (519 LGG and 163 GBM samples) and 208 normal brain tissue samples was analyzed using the “Differential Expression” module of GEPIA2.0 (Tang et al., 2017) (integrating TCGA and GTEx data).^1^ The “Survival Analysis” module of GEPIA2.0 was used to conduct a survival analysis of glioma patients with low and high expression of CLU. The “Correlation Analysis” module of GEPIA2.0 and the “Gene_Corr” module of TIMER2.0 (Li et al., 2020)^2^ were used to conduct a correlation analysis between CLU and its related genes. Protein-protein interaction (PPI) networks were constructed using the STRING (RRID:SCR_005223), and hub genes were identified by calculating network topological parameters such as degree centrality and betweenness centrality.

### Clinical specimen and ethical statement

Clinical glioma tissues and glioma pathologic diagnoses were obtained from Affiliated Hospital of Jianghan University. All samples were histologically diagnosed by pathologists based on the World Health Organization (WHO) classification for brain tumors. All patients provided written informed consent. The study protocol was approved by Ethics Committee of Jianghan University (JHDXKJLL2025–126). The specimens were immediately snap-frozen in liquid nitrogen after resection and stored at −*80*°C until further analysis.

### Quantitative real-time PCR assay

Total RNA was extracted from tissues or cells using a commercial RNA extraction kit (R30506, Shanghai yuanye Bio-Technology Co.) according to the manufacturer’s instructions. RNA (1 μg) was reverse transcribed to cDNA using the RevertAid RT Kit (K1691, Thermo Scientific). qPCR was performed on a real-time PCR system (CFX96 Deep Well Dx, Applied Biosystems) using TB Green^®^ Fast qPCR Mix (RR420A, Takara). The primer sequences (Sangon biotech) were as follow:

CLU Forward: 5′-AAACGAAGAGCGCAAGACAC-3′,

Reverse: 5′-TGTTTCAGGCAGGGCTTACA-3′;

BCL2L1 Forward: 5′-ACTCTTCCGGGATGGGGTAA-3′,

Reverse: 5′-TGTTTCAGGCAGGGCTTACA-3′;

ACTB Forward: 5′-ACTGGAACGGTGAAGGTGAC-3′,

Reverse: 5′-GGGACTTCCTGTAACAACGCA-3′.

The relative gene expression levels were calculated using the 2^–ΔΔCt^ method.

### Western blot

Cells or tissues were lysed in RIPA buffer containing protease and phosphatase inhibitors (P0013B, Beyotime). The protein concentration was determined by the BCA protein assay kit (AR0146, Abbkine). Equal amounts of protein (usually 10 μg) were separated by SDS-PAGE and transferred onto PVDF membranes. The membranes were blocked with 5% non-fat milk in TBST for 1 h at room temperature and then incubated with primary antibodies against the target proteins overnight at 4°C. After washing with TBST, the membranes were incubated with HRP-conjugated secondary antibodies for 1 h at room temperature. Enhanced Chemiluminescence (ECL) was used to detect chemiluminescent signals, which were quantified using ImageJ software (NIH, Bethesda, United States, RRID:SCR_003070). Bioassays were replicated three times.

### Immunofluorescence staining of tissue

Tissue samples were fixed in 4% paraformaldehyde for 24 h at 4°C, dehydrated through a graded series of ethanol solutions (70, 80, 95, and 100%), and then embedded in paraffin. Sections (5 μm thick) were cut using a microtome and mounted on poly-L-lysine-coated slides. The sections were dewaxed in xylene and rehydrated through a reverse ethanol series. Antigen retrieval was performed by boiling the sections in citrate buffer (pH 6.0) for 10 min in a microwave oven. After cooling, the sections were blocked with 10% normal goat serum in PBS for 1 h at room temperature. Subsequently, the sections were incubated with primary antibodies targeting Clusterin (1:1,000 dilution, R&D Systems, United States), Bcl-xl (1:1,000 dilution, ABclonal, China), and actin (1:1,000 dilution, Servicebio, China) diluted in 5% BSA-PBS overnight at 4°C. After washing three times with PBS, the sections were incubated with Alexa Fluor-conjugated secondary antibodies (1:200–1:500 dilution, Thermo Fisher Scientific, United States) for 1 h at room temperature in the dark. Nuclei were counterstained with DAPI (1 μg/mL, Thermo Fisher Scientific, United States) for 5 min. The stained sections were mounted with antifade mounting medium (Beyotime, China) and observed under a fluorescence microscope (Carl Zeiss AG, German). Bioassays were replicated three times.

### Cell lines and cell culture

Human cell lines, including U87, U251 (RRID:CVCL_0021), SW1783 and 293T (RRID:CVCL_0063) were obtained from Flight Biotech. The cells were cultured in high-glucose DMEM (for U251, SW1783 and 293T) and MEM (for U87) supplemented with 10% fetal bovine serum (FBS) and 1% penicillin-streptomycin at 37°C in a humidified atmosphere containing 5% CO_2_. The cells were passaged when they reached 80–90% confluence.

### Lentivirus and plasmid transfection

Lentiviral vectors (pVSVG-PATagRFP, Addgene) carrying the target genes or shRNAs were produced by co-transfecting the lentiviral packaging plasmids (psPAX2, Addgene) and the transfer plasmids into 293T cells using PEI transfection reagent. The supernatant containing the lentivirus was collected 48–72 h after transfection, filtered, and concentrated. Cells were infected with the lentivirus in the presence of polybrene. For plasmid transfection, cells were transfected with the plasmids using PEI according to the manufacturer’s instructions.

### Wound healing assay

Cells were seeded in 6 well plates and grown to confluence. A scratch was made in the cell monolayer using a sterile 200 μL pipette tip. The detached cells were removed by washing with PBS, and fresh medium was added. The wound closure was photographed at 0, 24, and 48 h using an inverted microscope. The migration distance was measured using image analysis software, and the relative migration rate was calculated.

### MTT assay

Cells were seeded in 96 well plates at a density of 2,000 cells per well. After plasmid transfection for 24 h, 10 μL of MTT reagent was added to each well, and the plates were incubated at 37°C for 4 h. The absorbance at 570 nm was measured using a microplate reader. The cell viability was calculated as a percentage of the control group.

### 5-ethynyl-2’-deoxyuridine cell proliferation assay

Cell proliferation rates were measured by the BeyoClick™ EdU Cell Proliferation Kit (Beyoteme, #C0078S; China). Cells were incubated with 500 μL of 2X EdU working solution (20μM) for 2 h at 37°C. Cells were fixed in 4% paraformaldehyde for 15 min, permeabilized with 0.3% Triton X-100 for 10 min, and incubated with the click reaction solution for 30 min. The representative images and the cell proliferation rate was analyzed by flow cytometry (Becton, Dickinson and Company, United States).

### Transwell migration assays

Glioma cells were added to the top chamber in serum-free media. The bottom chamber was filled with 10% FBS DMEM. After 24 h of incubation, the top chamber cells were removed using a cotton swab, and the membrane was fixed in 4% paraformaldehyde for 15 min and stained with crystal violet for 15 min. Five fields of adherent cells in each well were photographed randomly.

### Flow cytometric analysis of apoptosis

Cells were harvested, washed twice with cold PBS, and resuspended in binding buffer at a concentration of 1 × 10^6^ cells/mL. Annexin V-APC and 7-AAD were added to the cell suspension according to the manufacturer’s instructions (Annexin V-APC/7-AAD Apoptosis Kit, Multi sciences). The cells were incubated in the dark at room temperature for 15 min and then analyzed by flow cytometry (Becton, Dickinson and Company, United States). The percentage of apoptotic cells (Annexin V-APC-positive/7-AAD-negative and Annexin V-APC-positive/7-AAD-positive) was determined using FlowJo (RRID:SCR_008520) software (Tree Star, United States).

### Measuring mitochondrial membrane potential

ΔΨM was detected using JC-1 Mitochondrial Membrane Potential Assay Kit (Shanghai Yeasen Biotechnology Co., Ltd., Shanghai, China). ΔΨm was detected using JC-1 MMP assay kits (Shanghai Yeasen Biotechnology Co., Ltd., Shanghai, China). The cells were incubated with 1 × JC-1 staining working solution for 20 min at 37°C after different treatment. The cells were then washed twice with dilution buffer and imaged using an inverted fluorescence microscope (OLYMPUS, Japan). The relative ratio of red to green fluorescence intensity was acquired as the level of ΔΨM depolarization.

### Tumor xenograft in nude mouse

Male BALB/c nude mice (4 weeks old) were purchased from Beijing HFK Bioscience Co., Ltd. Animals were assigned to experimental groups using simple randomization. Cells (1×10^6^ cells in 100 μL of PBS) were subcutaneously injected into the right flank of the nude mice. The tumor volume was measured every 5 days using a caliper, and the volume was calculated using the formula V = 0.5 × length × width^2^. After a certain period of time, the mice were sacrificed, and the tumors were dissected, weighed, and processed for further analysis. The study protocol was approved by Ethics Committee of Jianghan University (JHDXLL2025–012).

### Orthotopic xenograft models

Four-week-old male BALB/c nude mice (Beijing HFK Bioscience Co., Ltd., China) were selected for the experiments. SW1783 cells (5 × 10^5^) transfected with an empty vector, CLU overexpression, or BCL2L1 knock-down were suspended in PBS and stereotactically implanted into the brains. The inoculation position was 2 mm lateral and 2 mm posterior to the anterior fontanel. Bioluminescence imaging was used to detect intracranial tumor growth on days 7, 14 and 21. Tumor tissues were harvested at 21 days after implantation, fixed in formalin, embedded in paraffin, sectioned and incubated with antibodies. The study protocol was approved by Ethics Committee of Jianghan University (JHDXLL2025–012).

### Hematoxylin-eosin staining

Tumor tissues were fixed in 10% formalin, embedded in paraffin, and sectioned at 5 μm thickness. The sections were de-paraffinized, re-hydrated, stained with hematoxylin for 5 min, differentiated with hydrochloric acid-ethanol, and counterstained with eosin for 3 min. The stained sections were dehydrated, cleared, and mounted with neutral balsam. The histological features of the tumor tissues were observed under a light microscope.

### Immunohistochemistry staining

Paraffin-embedded tissue sections were de-paraffinized and re-hydrated. Antigen retrieval was performed by heating the sections in citrate buffer (pH 6.0) in a microwave oven. The sections were blocked with 5% BSA in PBS for 1 h and then were incubated with primary antibodies (rabbit anti-Ki67 antibody, ABclonal Biotechnology, China) at 4°C overnight. After washing with PBS, the sections were incubated with HRP-conjugated secondary antibodies for 1 h at room temperature. The color was developed using DAB substrate, and the sections were counterstained with hematoxylin. The stained sections were dehydrated, cleared, and mounted, and the staining intensity was evaluated under a light microscope. Bioassays were replicated three times.

### Statistical analysis

All statistical analyses were performed using GraphPad Prism software. The data are presented as the mean ± standard deviation (SD). The significance of differences between groups was determined by Student’s *t*-test for comparisons between two groups and one-way ANOVA followed by Tukey’s *post-hoc* test for comparisons among multiple groups. *P* < 0.05 was considered statistically significant.

## Data Availability

The original contributions presented in this study are included in this article/Supplementary material, further inquiries can be directed to the corresponding author/s.

## Footnotes

1
http://gepia2.cancer-pku.cn/

2
http://timer.cistrome.org/

## Supplementary material

Supplementary Figure S1 The protein level of CLU differed in different glioma cell lines. **(A)** CLU protein levels in U87, U251, and SW1783 cell line were analyzed using Western blot (*n* = 3 biologically independent samples per group). **(B)** The data are shown as mean ± SEM with one-way ANOVA test, ***p* < 0̃.01.

Supplementary Figure S2 The intervention efficiency of CLU in SW1783 and U251. **(A)** CLU mRNA expression levels in SW1783 cell line were analyzed. **(B)** The protein expression of CLU was detected by Western blot in the SW1783 cell line after CLU overexpression. **(C)** The mRNA of CLU in U251 cell line were detected by real-time quantitative PCR. **(D)** The protein expression of CLU was detected by Western blot in the U251 cell line after CLU knockdown. All the data (*n* = 3 biologically independent samples per group) are normalized to the vector control group and presented as mean ± SEM. Statistical analysis was performed using an unpaired two-tailed Student’s *t*-test, **p* < 0.05.

Supplementary Figure S3 Reduced expression of CLU in U87 restrained cells migration and growth. **(A)** CLU mRNA expression levels in U87 cell line were analyzed (*n* = 3 biologically independent samples per group). The data are shown as mean ± SEM with unpaired two-tailed Student’s *t*-test, ****p* < 0.001. **(B)** The protein expression of CLU in U87 cell line were detected by western blot (*n* = 3 biologically independent samples per group). The data are shown as mean ± SEM with unpaired two-tailed Student’s *t*-test, ****p* < 0.001. **(C)** Representative images of scratch test from U87 cell line with or without reduced CLU expression. **(D)** Cell migration rate was analyzed after CLU knockdown (*n* = 6 biologically independent samples per group). The data are normalized to vector group and are shown as mean ± SEM with unpaired two-tailed Student’s *t*-test, **p* < 0.05. **(E)** Cell grow rate after CLU was reduced expressed were analyzed by MTT (*n* = 5 biologically independent samples per group). The data are normalized to 24 h group and shown as mean ± SEM with two-way ANOVA test, ***p* < 0̃.01.

Supplementary Figure S4 Associations of CLU expression with other genes in glioma. **(A)** The protein-protein interaction (PPI) network of CLU and related genes was constructed based on the STRING database. Nodes represent proteins (gene products), and nodes of different colors represent different genes. The connections between nodes indicate the existence of interactions between proteins, and the color and thickness of these connections represent the type and confidence level of the interactions. **(B–I)** Correlation of CLU expression with BAX, BCL2L1, PRNP, HSPA5, XRCC6, APP, LRP2, and TTR using TIMER2.0. **(J–Q)** Correlation of CLU expression with BAX, BCL2L1, PRNP, HSPA5, XRCC6, APP, LRP2, and TTR using GEPIA 2.0.

Supplementary Figure S5 **(A)** BCL2L mRNA levels were analyzed using real-time fluorescence quantitative PCR in the SW1783 cell line after CLU overexpression (*n* = 3 biologically independent samples per group).

Supplementary Figure S6 The intervention efficiency of CLU and BCL2L1 in SW1783 cell line. **(A)** The protein expression of CLU and Bclxl in SW1783 cell line were detected by western blot. **(B)** The data (*n* = 3 biologically independent samples per group) are shown as mean ± SEM with one-way ANOVA test, **p* < 0.05, ***p* < 0.01, ****p* < 0̃.01.

Supplementary Figure S7 The intervention efficiency of CLU and BCL2L1 in SW1783 cell line inoculated on the skin of nude mice. **(A)** The protein expression of CLU and Bclxl from tumor tissues were detected by western blot. **(B)** The data (*n* = 3 biologically independent samples per group) are shown as mean ± SEM with one-way ANOVA test, **p* < 0.05, ***p* < 0.01, ****p* < 0̃.01.

Supplementary Figure S8 BCL2L1 down regulation inhibits tumor formation promoted by CLU in nude mice. **(A)** Tumor volume was measured every 3 days for 21 days following subcutaneous inoculation of the SW1783 cell line in nude mice (*n* = 5 biologically independent samples per group). Data are presented as mean ± SEM and analyzed using a two-way ANOVA test. Statistical significance is indicated as follows: **p* < 0.05, ****p* < 0.001 for empty vector vs. CLU-OE; ^###^*p* < 0.001 for CLU-OE vs. BCL2L1-sh; ^$$^*p* < 0.01 for empty vector vs. BCL2L1-sh. **(B)** Representative images of nude mice with tumors. **(C)** Tumor weight was measured (*n* = 5 biologically independent samples per group) and presented as mean ± SEM. Statistical analysis was performed using a one-way ANOVA test, **p* < 0.05, ****p* < 0.001. **(D)** Histopathological features of the tumors were assessed using hematoxylin and eosin (H&E) staining. **(E)** The number of proliferating cells (Ki67-positive) was analyzed by immunohistochemistry. Data (*n* = 5 biologically independent samples per group) are presented as mean ± SEM and analyzed using a one-way ANOVA test, ***p* < 0.01, ****p* < 0.001.
