# Supplementary material for: Clusterin facilitates glioma progression via BCL2L1-dependent regulation of apoptotic resistance
Source: Front Mol Neurosci. 2025 Jun 18;18:1596021. doi: 10.3389/fnmol.2025.1596021 (PMC12216978; doi:10.3389/fnmol.2025.1596021)
Supplement: Supplementary Figure S1 — The protein level of CLU differed in different glioma cell lines. (A) CLU protein levels in U87, U251, and SW1783 cell line were analyzed using Western blot (n = 3 biologically independent samples per group). (B) The data are shown as mean ± SEM with one-way ANOVA test, **p < 0̃.01. [file Data_Sheet_1.docx]

**Clusterin Facilitates Glioma Progression via BCL2L1-Dependent Regulation of Apoptotic Resistance**

**Supplementary Figures**


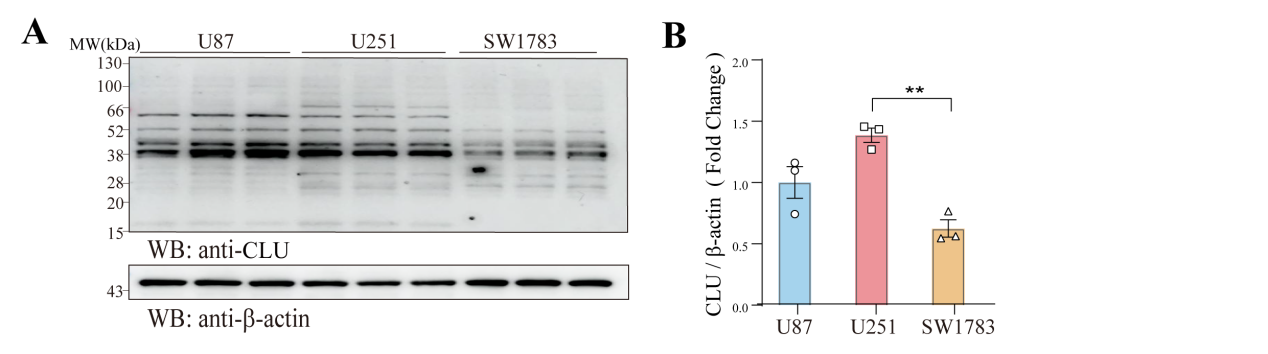


**Supplementary Figure 1: The protein level of CLU differed in different glioma cell lines.**

1. CLU protein levels in U87, U251 and SW1783 cell line were analyzed using Western blot (n=3 biologically independent samples per group). **B.** The data are shown as mean ± SEM with one-way ANOVA test, ***p*< 0.01.

**
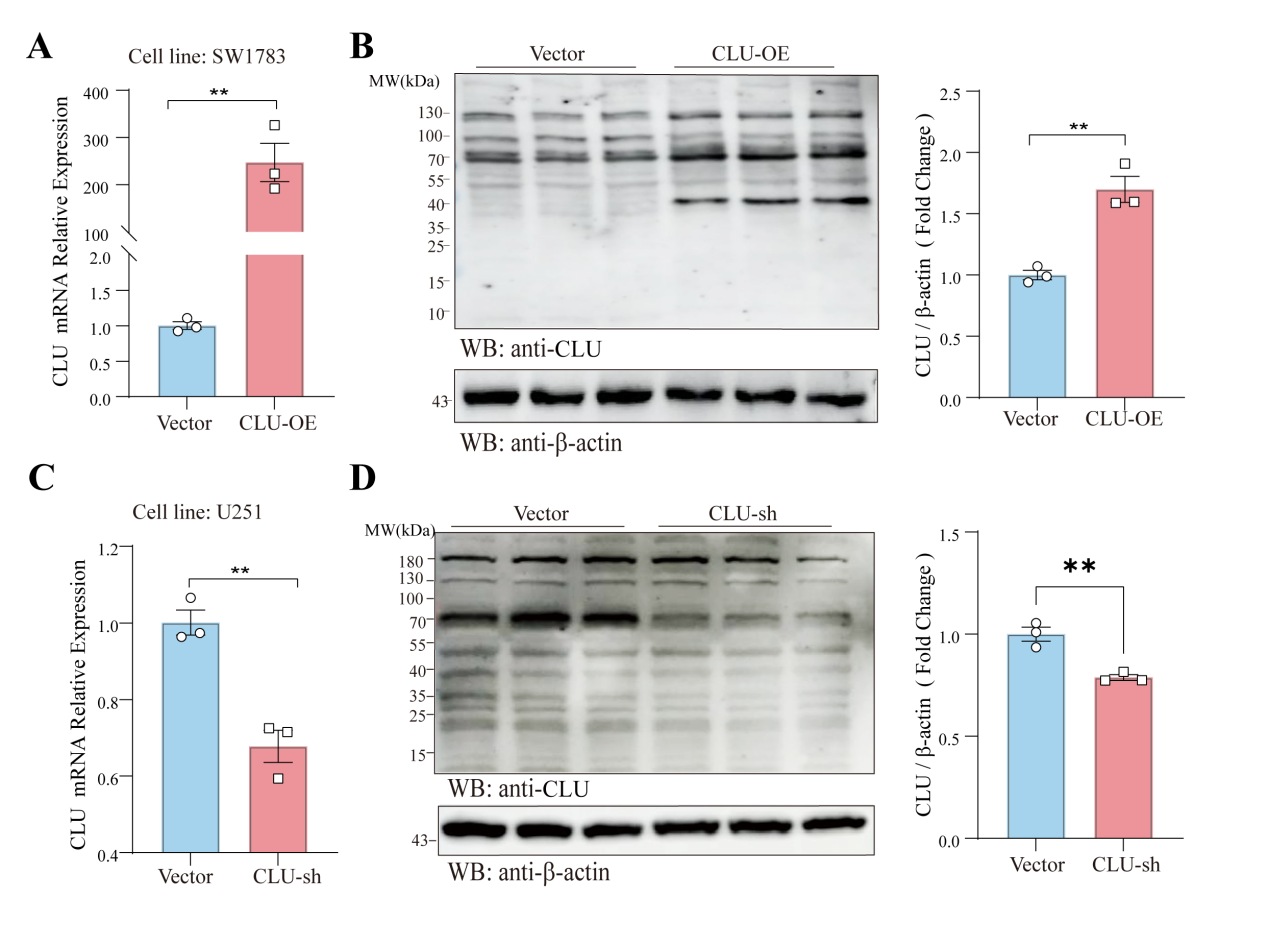
**

**Supplementary Figure 2: The intervention efficiency of CLU in SW1783 and U251.**

**A.** CLU mRNA expression levels in SW1783 cell line were analyzed. **B.** The protein expression of CLU was detected by Western blot in the SW1783 cell line after CLU overexpression. **C.** The mRNA of CLU in U251 cell line were detected by real-time quantitative PCR. **D.** The protein expression of CLU was detected by Western blot in the U251 cell line after CLU knockdown. All the data (n=3 biologically independent samples per group) are normalized to the vector control group and presented as mean ± SEM. Statistical analysis was performed using an unpaired two-tailed Student’s t-test, **p*<0.05.

**
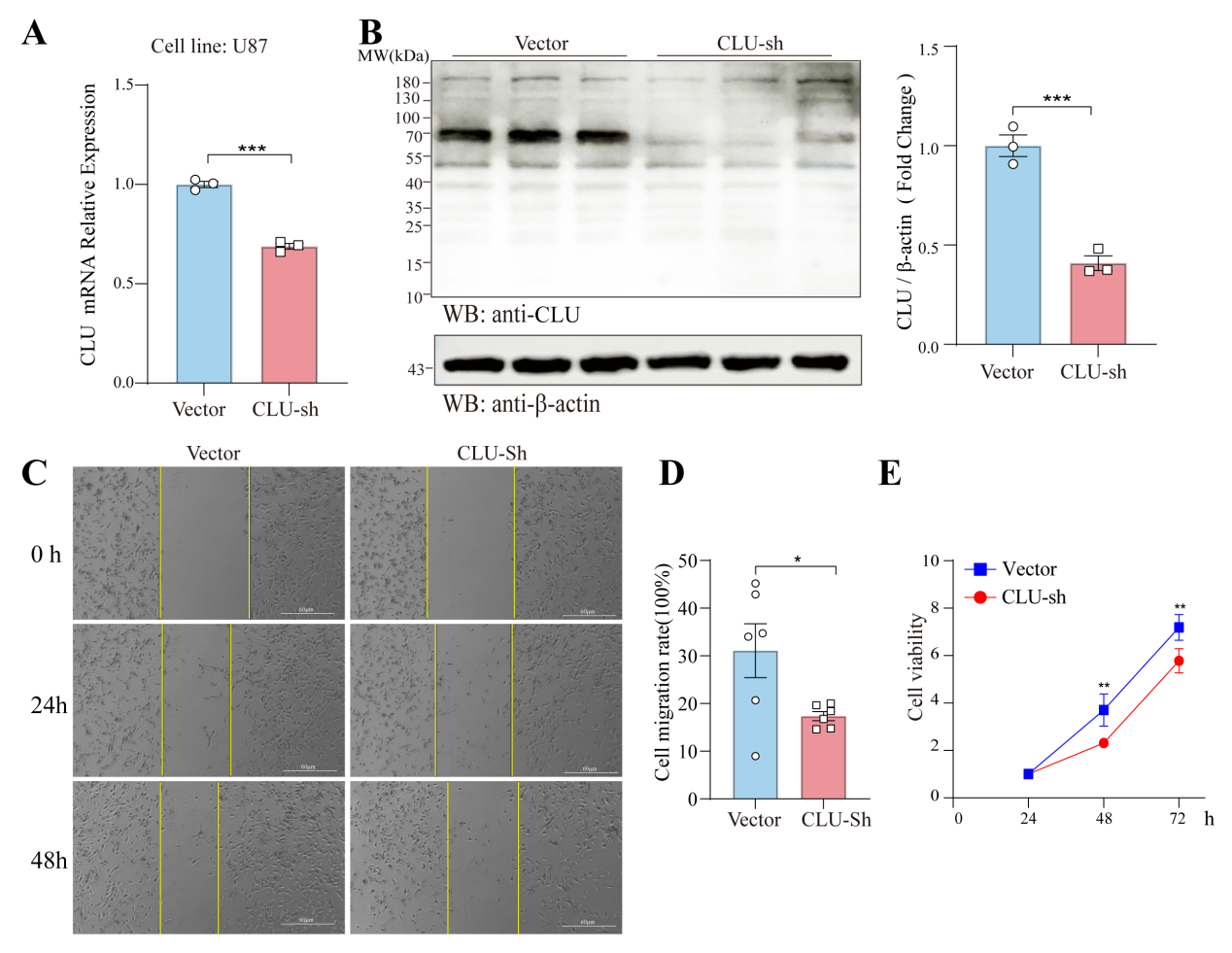
**

**Supplementary Figure 3: Reduced expression of CLU in U87 restrained cells migration and growth.**

**A.** CLU mRNA expression levels in U87 cell line were analyzed. (n=3 biologically independent samples per group). The data are shown as mean ± SEM with unpaired two-tailed Student’s t-test, ****p*< 0.001. **B.** The protein expression of CLU in U87 cell line were detected by western blot (n=3 biologically independent samples per group). The data are shown as mean ± SEM with unpaired two-tailed Student’s t-test, ****p*< 0.001. **C.** Representative images of scratch test from U87 cell line with or without reduced CLU expression. **D.** Cell migration rate was analyzed after CLU knockdown (n=6 biologically independent samples per group). The data are normalized to vector group and are shown as mean ± SEM with unpaired two-tailed Student’s t-test, **p*< 0.05. **E.** Cell grow rate after CLU was reduced expressed were analyzed by MTT (n=5 biologically independent samples per group). The data are normalized to 24h group and shown as mean ± SEM with two-way ANOVA test, ***p*< 0.01.

**
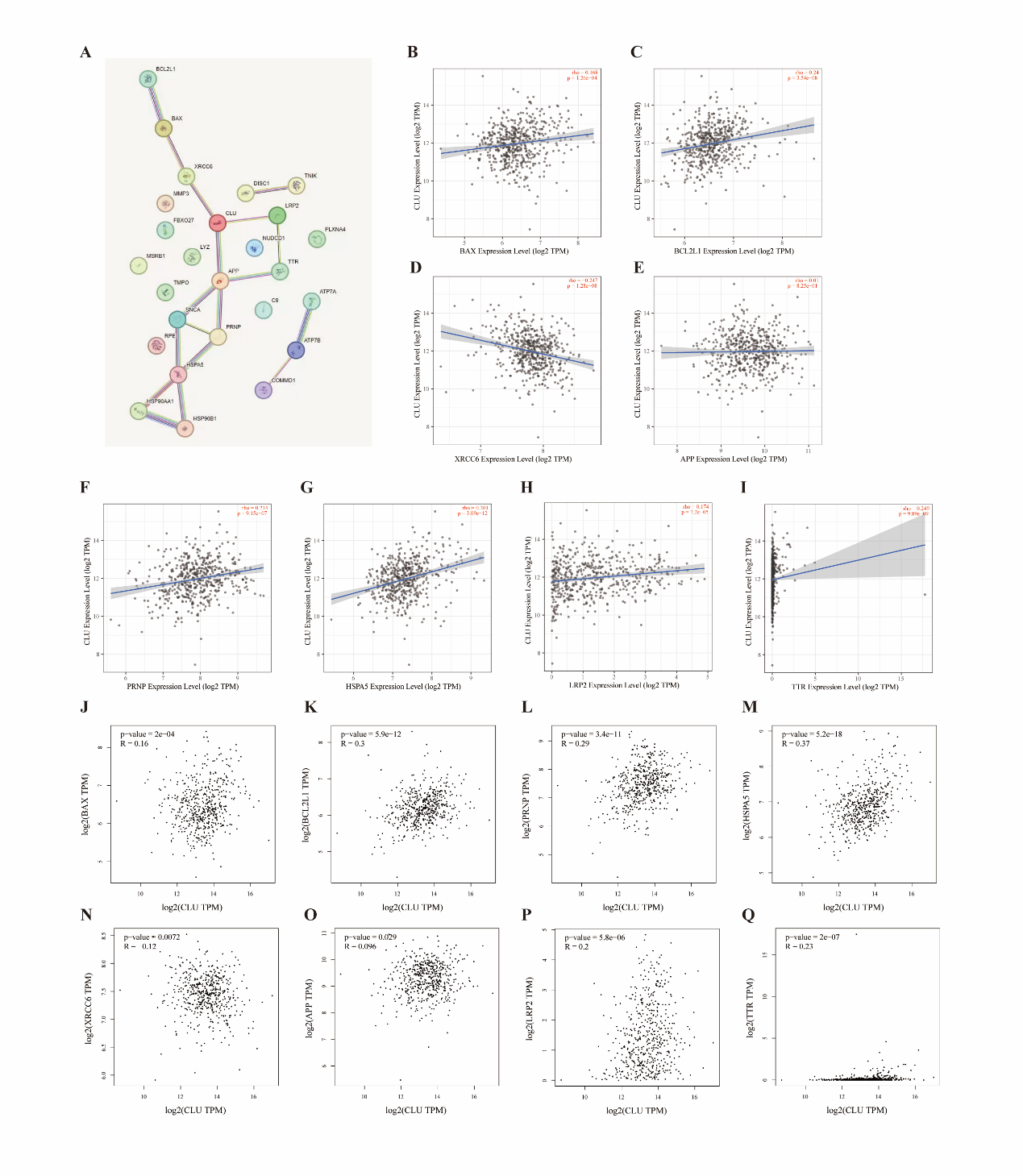
**

**Supplementary Figure 4: Associations of CLU expression with other genes in glioma.**

1. The protein - protein interaction (PPI) network of CLU and related genes was constructed based on the STRING database. Nodes represent proteins (gene products), and nodes of different colors represent different genes. The connections between nodes indicate the existence of interactions between proteins, and the color and thickness of these connections represent the type and confidence level of the interactions. **B-I.** Correlation of CLU expression with BAX, BCL2L1, PRNP, HSPA5, XRCC6, APP, LRP2 and TTR using TIMER2.0. **J-Q.** Correlation of CLU expression with BAX, BCL2L1, PRNP, HSPA5, XRCC6, APP, LRP2 and TTR using GEPIA 2.0.


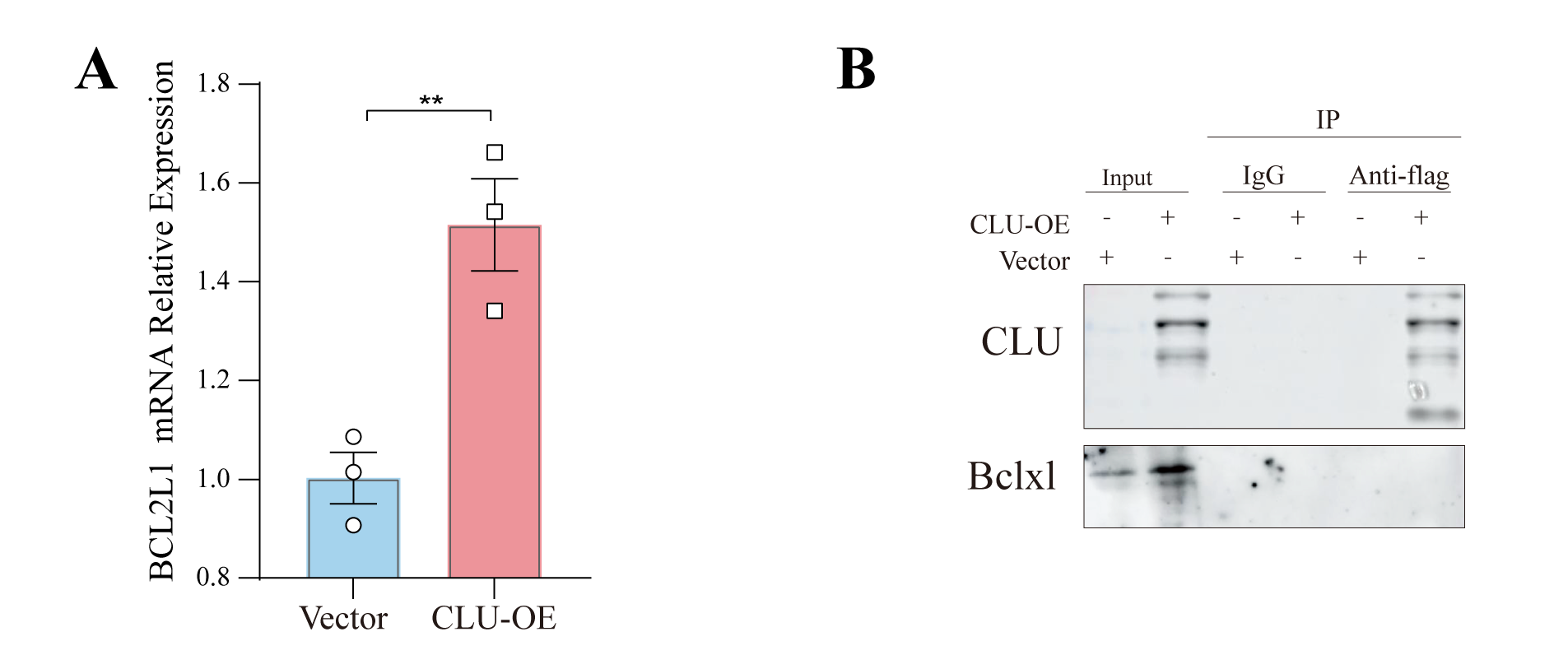


**Supplementary Figure 5: A**. BCL2L mRNA levels were analyzed using real-time fluorescence quantitative PCR in the SW1783 cell line after CLU overexpression (n=3 biologically independent samples per group).


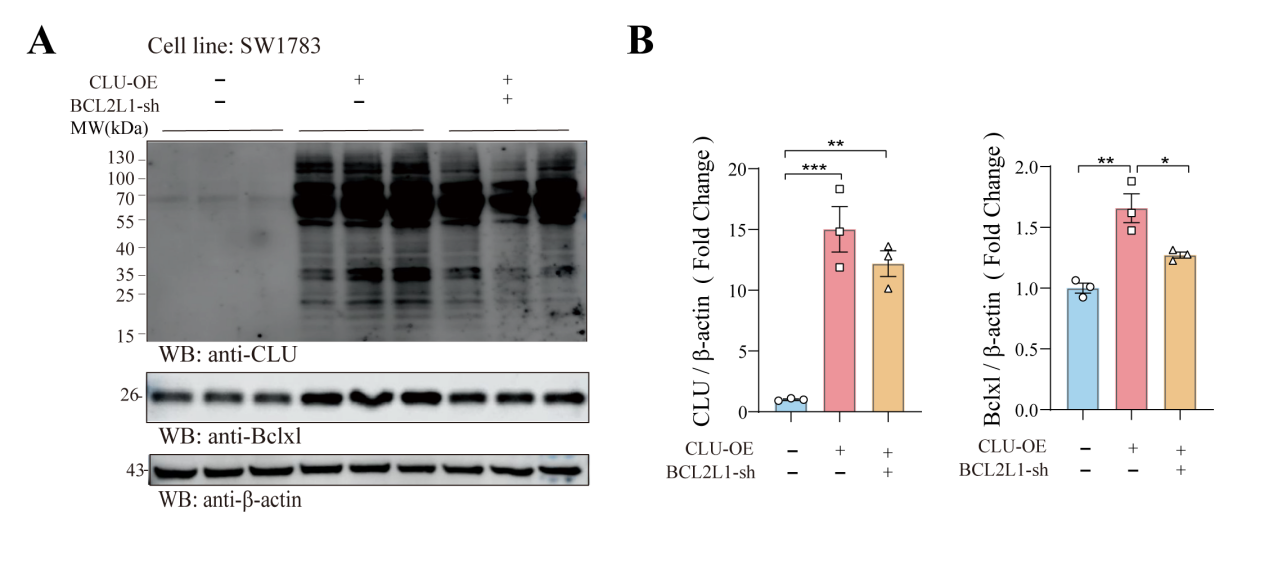


**Supplementary Figure 6: The intervention efficiency of CLU and BCL2L1 in SW1783 cell line.**

**A.** The protein expression of CLU and Bclxl in SW1783 cell line were detected by western blot. **B.** The data (n=3 biologically independent samples per group) are shown as mean ± SEM with one-way ANOVA test, **p*< 0.05, ***p*< 0.01, ****p*< 0.01.


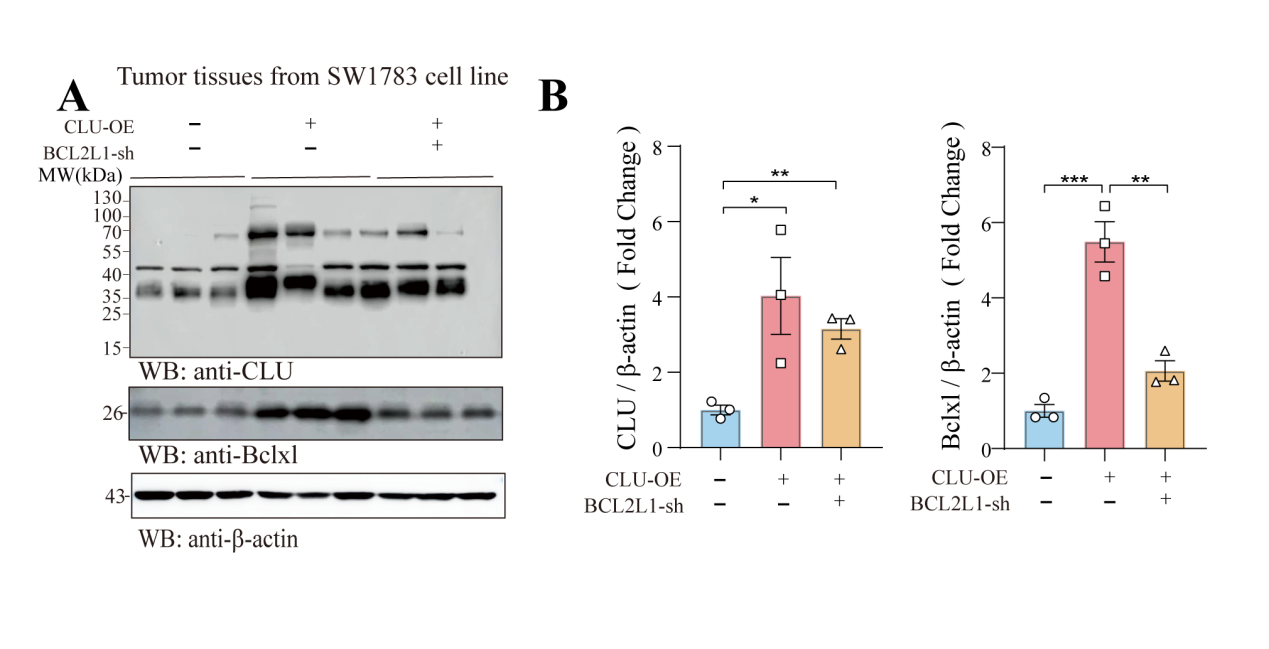


**Supplementary Figure 7: The intervention efficiency of CLU and BCL2L1 in SW1783 cell line inoculated on the skin of nude mice.**

**A.**The protein expression of CLU and Bclxl from tumor tissues were detected by western blot. **B.** The data (n=3 biologically independent samples per group) are shown as mean ± SEM with one-way ANOVA test, **p*< 0.05, ***p*< 0.01, ****p*< 0.01.


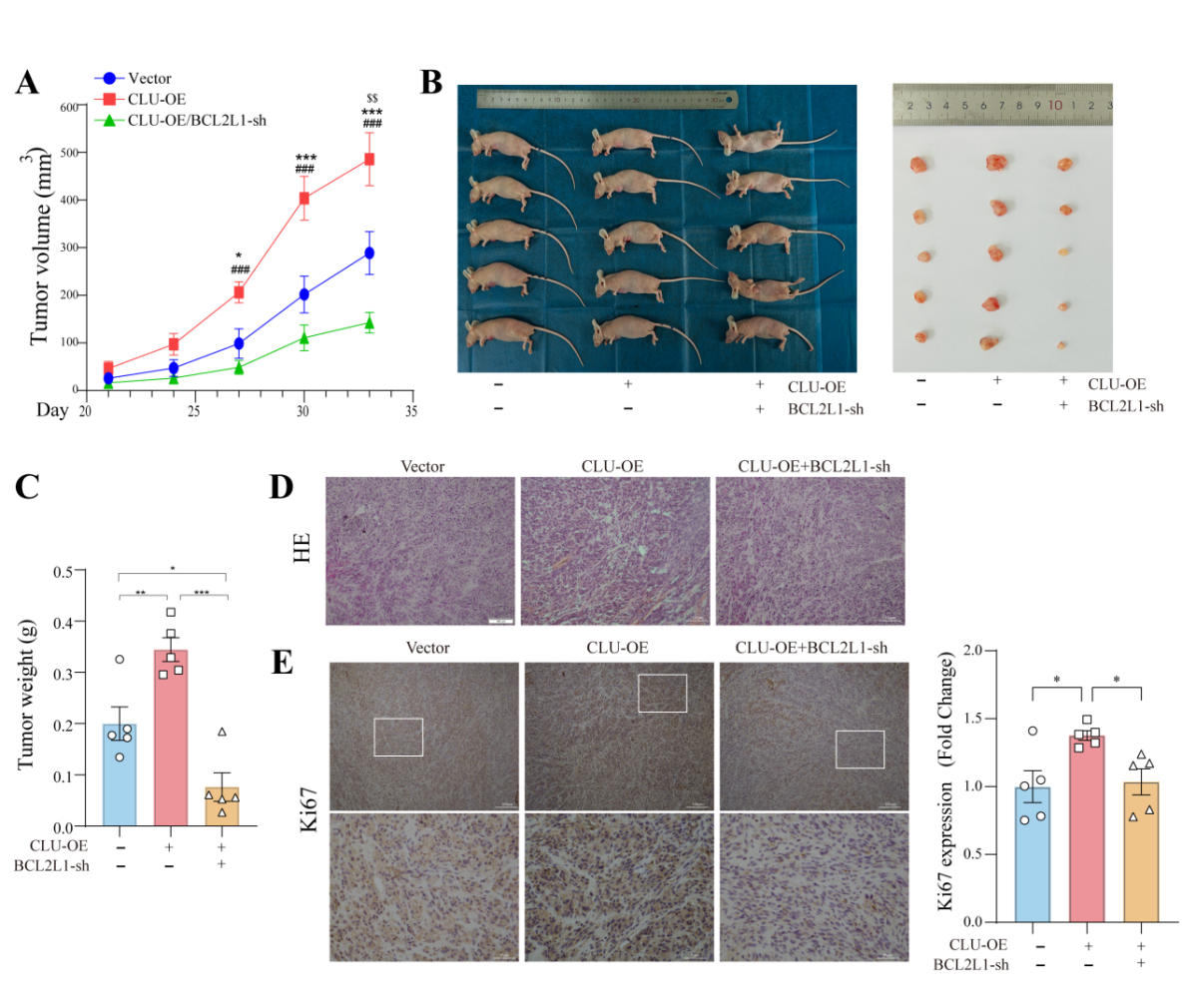


**Supplementary Figure 8: BCL2L1 down regulation inhibits tumor formation promoted by CLU in nude mice.**

**A**. Tumor volume was measured every 3 days for 21 days following subcutaneous inoculation of the SW1783 cell line in nude mice (n=5 biologically independent samples per group). Data are presented as mean ± SEM and analyzed using a two-way ANOVA test. Statistical significance is indicated as follows: **p*<0.05, ****p*<0.001 for empty vector vs. CLU-OE; ^###^*p*<0.001 for CLU-OE vs. BCL2L1-sh; ^$$^*p*<0.01 for empty vector vs. BCL2L1-sh. **B**. Representative images of nude mice with tumors. **C**. Tumor weight was measured (n=5 biologically independent samples per group) and presented as mean ± SEM. Statistical analysis was performed using a one-way ANOVA test, **p*<0.05, ****p*<0.001. **D**. Histopathological features of the tumors were assessed using hematoxylin and eosin (H&E) staining. **E**. The number of proliferating cells (Ki67-positive) was analyzed by immunohistochemistry. Data (n=5 biologically independent samples per group) are presented as mean ± SEM and analyzed using a one-way ANOVA test, ***p*<0.01, ****p*<0.001.
